# Couverture vaccinale et facteurs associés à la non complétude vaccinale des enfants de 12 à 23 mois du district de santé de Djoungolo-Cameroun en 2012

**DOI:** 10.11604/pamj.2014.17.91.2792

**Published:** 2014-02-04

**Authors:** Simon Franky Baonga Ba Pouth, Ditu Kazambu, Dieula Delissaint, Marie Kobela

**Affiliations:** 1 Central African Field Epidemiology And Laboratory Training Program, Université de Yaoundé 1, Cameroun; 2 Cellule de Supervision, Suivi et Evaluation, Délégation Régionale de la Santé Publique du Centre, Yaoundé, Cameroun; 3 Groupe Technique Central du Programme Elargi de Vaccination, Yaoundé, Cameroun; 4 Centre mère et enfant de la Fondation Chantal Biya, Yaoundé, Cameroun; 5 Faculté de Médecine et des Sciences Biomédicales de l'Université de Yaoundé 1, Cameroun

**Keywords:** Couverture vaccinale, facteurs associés, PEV, district, Cameroun, immunization coverage, associated factors, EPI, district, Cameroon

## Abstract

**Introduction:**

En 2011 le district de santé de Djoungolo a connu deux épidémies de rougeole avec un taux de complétude vaccinale de 69% selon les données du district. L'objectif de cette étude était de déterminer la couverture vaccinale et les facteurs associés à la non complétude vaccinale des enfants de 12 à 23 mois du district de Djoungolo en 2012.

**Méthodes:**

Une étude transversale à base communautaire a été menée en 2012. Au total 210 mères / nourrices d'enfants de 12 à 23 mois du district de Djoungolo, sélectionnées selon la méthode OMS de sondage en grappe 30 X 7, ont été interrogées sur les vaccins reçus par l'enfant avant l’âge d'un an et les raisons de non vaccination à l'aide d'un questionnaire structuré. Une analyse de régression logistique multivariée de type backward a été faite pour les variables ayant obtenu une valeur p < 0,2 à l'analyse univariée en utilisant le logiciel EPI Info version 3.5.3. Une association était significative lorsque p < 0,05.

**Résultats:**

La complétude vaccinale était de 64,3%, variant de 85,7% pour le BCG à 66,2% pour le Vaccin anti rougeoleux. La régression logistique multivariée a montré que les mères qui avaient peur des effets secondaires (P=0,0454), qui ne connaissaient pas l'importance de la vaccination (P=0,0139), qui avaient connu des occasions manquées de vaccination (P=0,0055), qui mettaient plus d'une heure pour vacciner leur enfant (P=0,0005) et qui ne maitrisaient pas le calendrier de vaccination (P=0,00001) étaient significativement associées à la non complétude vaccinale des enfants.

**Conclusion:**

La couverture vaccinale du district est en deçà des objectifs. Pour l'améliorer nous recommandons le renforcement de l’éducation des parents et une réorganisation des services de vaccination.

## Introduction

La vaccination est reconnue comme une des mesures les plus efficaces pour prévenir la mortalité, la morbidité et les complications des maladies infectieuses chez les enfants. On estime qu'environ 3 millions de décès sont évités chaque année dans le monde grâce à la vaccination et qu'en plus, elle permet chaque année d’éviter près de 750 000 enfants de souffrir de sérieux handicaps physiques, mentaux ou neurologiques [1].

En Mai 1974, l´Organisation mondiale de la Santé (OMS) a lancé un programme de vaccination mondiale, connu sous le nom de Programme Elargi de vaccination (PEV), comme l´une des interventions de santé publique majeure pour prévenir la morbidité et la mortalité infantiles. Le PEV vise à vacciner les enfants du monde entier pour prévenir les maladies, diminuer les invalidités et les décès dus aux maladies évitables par la vaccination [2].

La Vision et la Stratégie Mondiale pour la Vaccination 2006 - 2015, élaborée par l'OMS et l'UNICEF et adopté par la 56ème session du Comité régional de l'OMS pour l'Afrique envisage un monde dans lequel chaque enfant, adolescent et adulte dispose d'un accès équitable aux services de vaccination. Elle recommande également que l'on atteigne: « Un taux de couverture vaccinale national d'au moins 90% (dans tous les pays) et d'au moins 80% dans chaque district (ou unité administrative équivalente) vers l'an 2010 sinon plus tôt » [3].

Au Cameroun, le PEV du Ministère de la santé publique a élaboré et adopté un plan pluri annuel complet 2007 - 2011 du programme élargi de vaccination. Ce plan s’était fixé un objectif de couverture vaccinale pour tous les antigènes du PEV de routine y compris la Vitamine A d'au moins 88% au niveau national ceci en suivant le calendrier de vaccination [4].

Le district de santé de Djoungolo est le plus important district de santé de la région du Centre en termes de population. Selon les données administratives du district, sa couverture vaccinale était de 90% en 2009, 87% en 2010 et 69% en 2011; en cette année 2011, le district a connu deux épidémies de rougeole; la première flambée de cas a eu lieu d'Avril à Juin 2011 et a fait 23 cas avec 01 décès et la deuxième flambée de Novembre à Décembre 2011 avec 17 cas et 0 décès [5].

Malgré ces épidémies et cette couverture vaccinale administrative basse, aucune étude à notre connaissance n'a été conduite dans ce district pour déterminer la couverture vaccinale de ce district à l'aide d'une méthode standardisée d'une part et les raisons de non vaccination des enfants.

Cette étude vient donc combler ce gap. L'objectif de cette étude était de déterminer la couverture vaccinale de routine et les facteurs associés à la non complétude de la vaccination des enfants de 12 à 23 mois dans le district de santé de Djoungolo en 2012 dans le but de contribuer à la mise en place de stratégies spécifiques pour l'atteinte des objectifs de couverture vaccinale et de prévenir l'apparition de nouvelles flambées de maladies évitables par la vaccination dans ce district.

## Méthodes

### Cadre de l’étude

Cette étude a été menée dans les ménages du district de santé de Djoungolo, un des six districts de santé de la ville de Yaoundé comportant 90 quartiers répartis dans onze aires de santé. Il couvre une superficie 73 km^2^ pour une population estimé à 658 325 habitants en 2012. La population des enfants de 0 à 11 mois, cible du PEV est de 26 333 [6].

### Schéma de l’étude

Nous avons mené une étude transversale à base communautaire, entre février et août 2012, en administrant un questionnaire structuré préalablement prétesté à 210 mères / nourrices d'enfants de 12 à 23 mois du district de santé de Djoungolo (7 par quartier), choisies dans 30 quartiers selon la méthode de sondage en grappe type 30X7 de l'OMS [7]. Nos critères d'inclusion étaient d’être résidants du district au moment de l’étude et de l'avoir été en 2011.

Les onze aires de santé ont toutes été retenues comme unités primaires. 30 quartiers ont été tirés au sort sur les 90 en tenant compte de la taille de la population dans chaque aire de santé (allocation proportionnelle). Dans chaque quartier ou village étudié, les ménages à visiter ont été choisis au hasard. A partir du centre du village, nous avons numéroté toutes les maisons s'y trouvant; le premier ménage à visiter était choisi en tirant au sort. Dans les ménages visités, seul un enfant éligible, le plus jeune, était enquêté. La seconde concession visitée était celle qui se trouvait immédiatement à droite, en sortant de la première. Ainsi l’évolution s'est faite de proche en proche jusqu´à la complétude du nombre d'enfants à recruter pour le quartier.

### Outils et technique de collecte des données

A l'aide d'un questionnaire structuré préalablement testé, les mères/nourrices ont été interrogées sur les vaccins reçus par l'enfant avant l’âge d'un an et les raisons de non vaccination. Les informations collectées incluaient les caractéristiques socio démographiques, les facteurs liés au système de vaccination et les connaissances, attitudes et pratiques des mères sur la vaccination. Les vaccins reçus par l'enfant étaient obtenus à partir de la carte de vaccination ou à travers l'histoire vaccinale de l'enfant relatée par la mère.

### Définition des variables

Nos variables dépendantes étaient la complétude vaccinale et la couverture vaccinale spécifique par antigène des enfants de 12 à 23 mois. Un enfant était dit complètement vacciné s'il avait reçu les 06 doses de vaccins suivants avant l’âge de 12 mois: BCG, DTC-HépB1+Hib1, DTC-HépB1+Hib2, DTC-HépB1+Hib3, VAR et l'Anti Amaril selon la carte de vaccination et/ou les déclarations de la mère /nourrice. La couverture vaccinale par antigène était défini par le rapport entre le nombre d'enfants de 12 à 23 mois ayant reçu cet antigène avant l’âge de 12 mois sur le nombre total d'enfants de 12 à 23 mois enquêtés.

Les variables indépendantes étaient les fréquences des différentes modalités des caractéristiques socio démographiques de l’échantillon, des facteurs liés au système de vaccination et des connaissances, attitudes et pratiques des mères vis-à-vis de la vaccination.

### Analyse des données

Les données ont été saisies dans une base de données et les analyses faites à l'aide du logiciel Epi Info 3.5.3. Les contrôles de saisie nous ont permis de minimiser les erreurs. L'analyse des facteurs associés à la non complétude vaccinale s'est faite selon un modèle de régression logistique multivariée de type « backward »: la première était une analyse univariée par régression logistique qui nous a permis d'obtenir des odds ratio bruts pour chacune de ces variables avec leurs intervalles de confiance à 95% et leurs valeurs P. Ensuite les variables ayant obtenu une valeur de p < 0,2 ont été toutes entrées dans un modèle de régression logistique multivariée pour contrôler les facteurs de confusion et de déterminer quelles caractéristiques sont des prédicteurs indépendants de l´état vaccinal de l´enfant. Une valeur P < 0,05 et un ratio de côte ajusté (AOR) avec son intervalle de confiance à 95% qui ne contient pas 1,00 était considéré comme significatif. La variable qui avait obtenu dans ce modèle n, la plus grande valeur P non significative (> 0,05), était sortie du modèle; on obtenait donc un modèle n-1 et ainsi de suite jusqu’à ce que toutes les variables du modèle n-x aient une valeur P < 0,05.

### Considérations éthiques

Cette étude a obtenu l'autorisation de la Faculté de Médecine de L'Université de Yaoundé 1 et des autorités sanitaires de la région du Centre pour être menée. Un consentement éclairé verbal était requis pour chaque participant avant l'administration du questionnaire. Pour les participants, un certain bénéfice était retiré du fait qu'ils étaient informés du retard vaccinal éventuel de leur enfant et des informations leur étaient données quant aux vaccins manquants.

## Résultats

### Caractéristiques sociodémographiques de la population d’étude

Deux cent dix mères/nourrices d'enfants de 12 à 23 mois ont été inclues dans cette étude. L’âge des répondants variait de 17 à 44 ans avec une moyenne de 27±6ans. La classe d’âge la plus représentée était celle des 25 à 34 ans (49,5%). Cent trente six (64,8%) mères/nourrices avaient un niveau d’étude au moins égale au niveau secondaire et quinze (7,1%) n’étaient jamais allés à l’école. Trente cinq (16,7%) vivaient seules alors que 175 (83,3%) étaient en couple. Chez celles qui étaient en couple, l'union libre était la forme la plus retrouvée (40,55%) suivie du mariage monogamique (32,4%); 59% étaient de religion catholique, suivi des protestants (22,4%) et des musulmans (11%); 40,5% des ménages avaient un revenu mensuel moyen compris entre 50 000 et 150 000 F cfa comme illustré dans le Tableau 1.

**Tableau 1:** Répartition des répondants selon certains caractéristiques socio démographiques, District de Santé de Djoungolo 2012

Variable	Catégories	Fréquence	(%)
Classe d’âge	≤ 24 ans	78	(37,2)
	25 à 34 ans	104	(49,5)
	≥ 35 ans	28	(13,3)
Niveau d'instruction	Aucun	15	(7,1)
	Primaire	59	(28,1)
	Secondaire	127	(60,5)
	Supérieur	9	(4,3)
Statut matrimonial	Mariée monogamique	68	(32,4)
	Marié polygamique	22	(10,4)
	Union libre	85	(40,5)
	Célibataire seule	33	(15,7)
	Divorcée/ Séparée	1	(0,5)
	Veuve	1	(0,5)
Religion	Catholique	124	(59,0)
	Protestant	47	(22,4)
	Musulman	23	(11,0)
	Pentecôtiste	8	(3,8)
	Témoins de Jéhovah	1	(0,5)
	Autres	7	(3,0)
Revenus moyens mensuels du ménage	< 50 000 F cfa	26	(12,4)
	50 000 à 150 000 F cfa	85	(40,5)
	> 150 000 F cfa	59	(28,1)
	Non Renseigné	40	(19,0)

Des 210 enfants inclus dans cette étude, 50% étaient de sexe masculin; leur âge variait de 12 à 23 mois avec une moyenne de 17 ± 3 mois et 68,6% étaient comptés parmi les trois premiers enfants de la fratrie.

### La couverture vaccinale des enfants de 12 à 23 mois dans le district de santé de Djoungolo

Des 210 enfants de 12 à 23 mois inclus dans cette étude, 140 (66,7%) avaient une carte de vaccination. La couverture vaccinale selon les cartes de vaccination était de 54,3%. Le Tableau 2 montre que selon la carte de vaccination et les déclarations des mères/ nourrices, 135 (64,3%) étaient complètement vaccinés, 67 (31,9%) étaient partiellement vaccinés et 8 (3,8%) n'avaient reçu aucun vaccin.

**Tableau 2:** Taux de complétude vaccinale des enfants de 12 à 23 mois dans le district de sante de Djoungolo en 2012

Variable	Catégories	Selon Carte		Selon Carte + Déclarations mères / nourrices	
		Fréquence	(%)	Fréquence	(%)
Disponibilité Carte de Vaccination	Oui	140	(66,7)	--	--
Enfant vacciné (Au moins ne dose)	Oui	140	(66,7)	202	(96,2)
Statut Vaccinal	Complètement vacciné	114	(54,3)	135	(64,3)
	Partiellement vacciné	26	(12,4)	67	(31,9)
	Aucun vaccin	--	--	8	(3,8)

% : pourcentage

**Figure 1:**
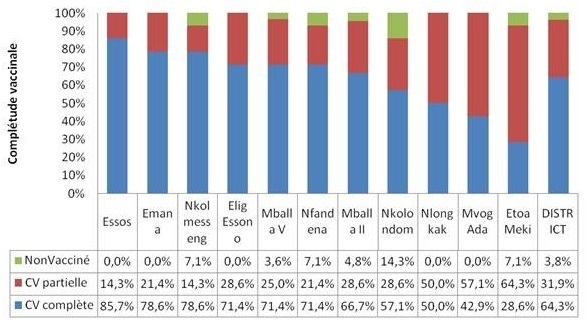
Taux de complétude vaccinale par aire de sante dans le district de sante de Djoungolo en 2012

Cette couverture variait de 28,6% dans l'aire de santé d'Etoa Meki à 85,7% dans l'aire de santé d'Essos. L'aire de santé de Nkolondom présentait la plus grande proportion d'enfant n'ayant reçu aucun vaccin (14,3%), suivi des aires de santé de Nkolmessseng, Nfandena et Etoa Meki (7,1%) comme illustré dans la Figure 1.

**Figure 2:**
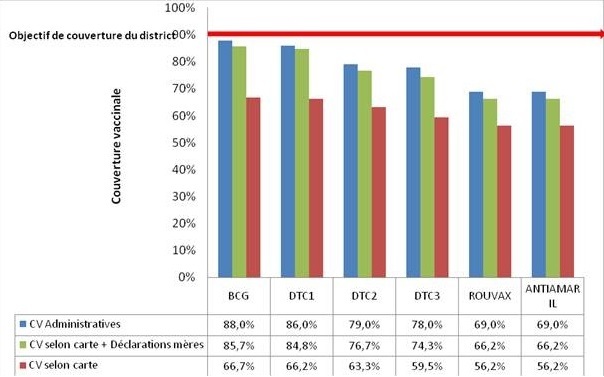
Taux de couverture vaccinale spécifique par antigène des enfants de 12 à 23 mois du district de sante de Djoungolo en 2012 selon les résultats de l'enquête et les données administratives

La Figure 2 montre que les couvertures vaccinales spécifiques par antigène selon la carte de vaccination et les déclarations des mères/nourrices étaient de: 85,7% (BCG), 84,8% (DTC-HépB+Hib1), 76,7% (DTC-HépB+Hib2), 74,3% (DTC-HépB+Hib3), 66,2% (Vaccin anti Rougeoleux) et 66,2% (Vaccin anti Amaril).

### Raisons de non vaccination des mères d'enfants de 12 à 23 mois non ou partiellement vaccinés du district de santé de Djoungolo en 2012

Soixante quinze mères/nourrices d'enfants de 12 à 23 mois non ou partiellement vaccinés ont donné les raisons de non vaccination de leur enfant.

Le manque d'information sur la vaccination (65,3%), les heures de vaccination inadaptées à la mère /nourrice (45,3%) et l'indisponibilité de la mère/nourrice (28,0%) étaient les trois raisons les plus fréquemment évoquées. Trois répondantes, toutes mères /nourrices d'enfants n'ayant reçu aucun vaccin ont déclaré que la vaccination était contraire à leurs convictions religieuses. Les heures de vaccination inadaptées étaient significativement associées aux mères d'enfants partiellement vaccinés (P=0,008) alors que les convictions religieuses contraires (P=0,0001) et la perception de l'inefficacité de la vaccination (P=0,001) étaient significativement associées aux mères d'enfants n'ayant reçu aucun vaccin comme illustré dans le Tableau 3.

**Tableau 3:** Fréquences des raisons de non vaccination évoquées par les mères d'enfants de 12 à 23 mois non ou partiellement vaccines du district de sante de Djoungolo en 2012

N°	Raisons de non vaccination	Chez les mères d'enfants partiellement vaccinés (n = 67)		Chez les mères d'enfant n'ayant reçu aucun vaccin (n = 8)		Total* (n = 75)				
		n	%	N	%	n	%	OR	95% IC	P value
1	Manque d'informations sur la vaccination (calendrier vaccination, dates rendez vous)	46	68,7	3	37,5	49	65,3	3,6587	0,6412 – 25,2409	0, 117
2	Heures de vaccination non adaptées à la mère	34	50,7	0	0,0	34	45,3	--	--	0,008
3	Indisponibilité de la mère/ nourrice	21	31,3	0	0,0	21	28,0	--	--	0,064
4	Enfant indisponible (maladie, voyage)	19	28,4	0	0,0	19	25,3	--	--	0,083
5	Eloignement du site de vaccination	17	25,4	2	25,0	19	25,3	1,0245	0,1981 7,9413	0,835
6	Enfant amené mais non vacciné	17	25,4	0	0,0	17	22,7	--	--	0,108
7	Peur des effets secondaires	15	22,4	0	0,0	15	20,0	--	--	0,137
8	Manque d'argent	12	17,9	0	0,0	12	16,0	--	--	0,195
9	Mauvais accueil par le personnel de santé	12	17,9	0	0,0	12	16,0	--	--	0,195
10	Vaccination non efficace	6	9,0	4	50,0	10	13,3	0,10	0,02 0,56	0,001
11	Convictions religieuses contraires à la vaccination	0	0,0	3	37,5	3	4,0	--	--	0,000
12	Autres	14	20,9	0	0,0	14	18,7	--	--	--

* Certains répondants ont donné plus d'une raison. (n= effectif ; OR = odds ratio ; 95% IC = Intervalle de confiance à 95% ; % = pourcentage)

### Prédicteurs indépendants de la non complétude vaccinale des enfants de 12 à 23 mois du district de santé de Djoungolo en 2012

Nous avons trouvé que les mères d'enfants de 12 à 23 mois du district de santé de Djoungolo qui avaient peur de la survenue des effets secondaires (P=0,0454), qui ne connaissaient pas l'importance de la vaccination (P=0,0139), qui n'avaient participé à aucune causerie éducative sur la vaccination en 2011 au centre de vaccination (P=0,0363), qui avaient connu au moins une occasion manquée de vaccination (P=0,005), qui mettaient plus d'une heure pour vacciner leur enfant (P=0,005) et qui ne maitrisaient pas le calendrier de vaccination (P=0,00001) étaient significativement associées à l'incomplétude vaccinale des enfants comme illustré dans le Tableau 4.

**Tableau 4:** Facteurs associés à l'incomplétude vaccinale des enfants de 12 à 23 mois du district de sante de Djoungolo en 2012, Résultat de la régression logistique multiple

Facteurs*	AOR	95%IC		P value
La crainte de la survenue des effets secondaires (OUI / NON)	3,7608	1,0275	13,7551	0,0454
La non participation à au moins une séance d'IEC sur la vaccination au centre de vaccination (OUI / NON)	2,3314	1,0555	5,1494	0,0363
La non connaissance de l'importance de la vaccination (OUI / NON)	4,4147	1,3514	14,4216	0,0139
Expérience occasion manquée (OUI / NON)	4,1928	1,5234	11,5340	0,0055
Délais d'attente au site de vaccination supérieur à 1h (PLUS 1H / MOINS 1H)	8,3892	2,5329	27,7778	0,0005
La non maitrise du calendrier de vaccination vaccinal (OUI / NON)	16,4148	4,8917	55,0824	0,0000

* Seuls les facteurs significatifs sont présentés dans ce tableau. AOR = ratio de côte ajusté; 95% IC = intervalle de confiance à 95%

## Discussion

### La complétude vaccinale

Notre étude a trouvé un taux de complétude vaccinale de 64,3% dans le district de santé de Djoungolo en 2012 avec 3,8% d'enfants non vaccinés. Ce résultat bien qu'inférieur aux données du district (69%) [5] montre une amélioration par rapport aux résultats de l'EDS-MICS 2011 (59,9% à Yaoundé en 2010) [8]. Mais l'objectif de couverture du district de 90% n'a pas été atteint et ceci pour aucun antigène. Le risque d’épidémies des maladies ciblées est donc important dans ce district.

Le taux de possession de la carte de vaccination était de 66,7%. Ce résultat est conforme à la plupart des enquêtes de couverture vaccinale réalisées au Cameroun [8–10] et en Afrique [11–16]. La sensibilisation et le renforcement de l'obligation de présentation de la carte de vaccination avant toute inscription de l'enfant à la maternelle pourrait améliorer le taux de détention des cartes de vaccination par les parents.

### Couverture vaccinale par Aire de santé

Nos résultats montrent que dans aucune aire de santé, l'objectif de complétude vaccinale de 90% n'a été atteint. La plus mauvaise performance a été retrouvée dans l'aire de santé d'Etoa Meki (28,6%), une aire de santé totalement urbaine. L'absence d'une formation sanitaire leader publique et la non réalisation des stratégies avancées par le district dans cette aire expliqueraient ces mauvaises performances. Les aires de santé rurale de Nkolmesseng et de Nkolondom ont obtenues des performances supérieures à certaines aires de santé urbaines (Nlongkak, Mvog ada, Etoa Meki); cette tendance à une meilleure complétude vaccinale des enfants en zone rurale par rapport à la zone urbaine a été soulignée par Wiysonge et al. dans une méta analyse de 24 enquêtes démographiques et de santé faites en Afrique au sud du sahara entre 2003 et 2010 [17].

Dans les aires de santé de Nkolmesseng, Mballa V, Mfandena, Nkolondom et Etoa Meki, des enfants « zéro dose », n'ayant reçu aucun vaccin du PEV avant leur première année de vie, ont été identifiés. l'EDS MICS 2011 avaient déjà retrouvé 4,5% d'enfants non vaccinés à Yaoundé [8]. Ces enfants représentent une menace pour l'immunité collective et le contrôle des maladies cibles dans ce district. Les facteurs associés à la non vaccination de ces enfants devront être identifiés et des stratégies spécifiques d'atteinte de ceux-ci mises en oeuvre.

### Facteurs associés à la non complétude vaccinale des enfants

Dans cette étude, les analyses multivariées réalisées nous ont permis de mettre en évidence une association statistiquement significative entre la crainte de la survenue des effets secondaires, la non connaissance de l'importance de la vaccination, l'expérience par la mère / nourrice d'une occasion manquée de vaccination, le délai d'attente au site de vaccination supérieur à 1 heure, la non maitrise par la mère/ nourrice du calendrier vaccinal et la non complétude vaccinale des enfants de 12 à 23 mois.

### La non maitrise par la mère/ nourrice du calendrier vaccinal

C'est le facteur prédictif indépendant le plus significativement associé à l'incomplétude vaccinale dans notre étude. Il a été cité comme la première cause de non vaccination par les mères d'enfants non ou partiellement vaccinés. Les parents ne maitrisent pas correctement à quel âge l'enfant doit commencer et terminer ses vaccins, ni même le nombre total de vaccins à prendre. Ce résultat rejoint ceux retrouvés au Nigéria en 2008 [18], au Sénégal en 2009 [19] et en Ethiopie en 2011 [20].

### Le délai d'attente au site de vaccination supérieur à 1 heure

Ce facteur est lié à l'organisation du poste et de la séance de la vaccination. Il avait déjà été retrouvé par l'enquête de couverture vaccinale de 2005 au Cameroun [9] comme la principale cause de non vaccination des enfants.

### L'expérience par la mère / nourrice d'une occasion manquée de vaccination

Le fait de ne pas administrer une dose d'antigène à un enfant lorsqu'il se présente à son rendez vous de vaccination pour une raison quelconque (« maladie de l'enfant », absence de vaccins, absence du vaccinateur,) a été trouvé significativement associé à la non complétude vaccinale des enfants dans le district de Djoungolo. Ce facteur avait déjà été décrit comme associé à la non vaccination des enfants par l'enquête de couverture vaccinale au Cameroun de 2005 [9] et aussi relevé par d'autres auteurs en Afrique au sud du Sahara [18–21].

### La non connaissance de l'importance de la vaccination

Les résultats de notre étude montrent que les parents qui ne connaissent pas l'importance de la vaccination ou alors ont des perceptions erronées ne montrent pas un grand engouement à vacciner leur enfant. Cette insuffisance d'information sur la vaccination a été décrite comme la deuxième cause de non vaccination par l'enquête de couverture vaccinale de 2005 au Cameroun [9].

### La crainte de la survenue des effets secondaires

Les parents qui ont eu ou connu un enfant ayant développé un effet secondaire modéré à grave après vaccination développent des craintes vis-à-vis des prochaines vaccinations. Ici une fois de plus, le système de santé est interpellé car la plupart de ces effets sont prévisibles. Les parents devraient donc être sensibilisés sur leur survenue éventuelle et la conduite à tenir. Ceci rentre dans le cadre global de l'insuffisance d'information des parents sur la vaccination, facteur décrit par d’ autres auteurs au Cameroun [9] et en Afrique au sud du Sahara [18–21].

### Limites de l’étude

La principale limite de notre étude était les biais de mémoire qui pouvait entrainer une mauvaise classification du statut vaccinal. Dans notre étude, seul 66,7% d'enfants possédaient une carte de vaccination. Les informations étaient donc complétées par les déclarations des mères qui étaient vérifiées par des questions croisées visant à préciser les dates, lieux de vaccination, le site d'administration, la dose reçu, la présence de la cicatrice (BCG). Les prestataires n'ont pas été considérés dans la recherche des facteurs associés à la complétude vaccinale. Des études ultérieures devront s'intéresser à cet aspect.

## Conclusion

La couverture vaccinale du district de santé de Djoungolo en 2012 est très en deçà des objectifs. Des aires de santé avec des enfants n'ayant jamais reçu aucune vaccination ont été identifiée. L'insuffisance de connaissance des mères sur la vaccination, les occasions manquées et les longs délais d'attente sont les facteurs prédictifs les plus importants de la non vaccination des enfants. Pour améliorer la couverture vaccinale dans ce district, nous recommandons le renforcement de l’éducation des mères sur la vaccination et une réorganisation des services de vaccination.
